# Using Poison Center Exposure Calls to Predict Methadone Poisoning Deaths

**DOI:** 10.1371/journal.pone.0041181

**Published:** 2012-07-19

**Authors:** Nabarun Dasgupta, Jonathan Davis, Michele Jonsson Funk, Richard Dart

**Affiliations:** 1 Department of Epidemiology, Gillings School of Global Public Health, University of North Carolina at Chapel Hill, North Carolina, United States of America; 2 Rocky Mountain Poison and Drug Center, Denver Health, Denver, Colorado, United States of America; 3 School of Medicine, University of Colorado, Denver, Colorado, United States of America; University of Louisville, United States of America

## Abstract

**Purpose:**

There are more drug overdose deaths in the Untied States than motor vehicle fatalities. Yet the US vital statistics reporting system is of limited value because the data are delayed by four years. Poison centers report data within an hour of the event, but previous studies suggested a small proportion of poisoning deaths are reported to poison centers (PC). In an era of improved electronic surveillance capabilities, exposure calls to PCs may be an alternate indicator of trends in overdose mortality.

**Methods:**

We used PC call counts for methadone that were reported to the Researched Abuse, Diversion and Addiction-Related Surveillance (RADARS®) System in 2006 and 2007. US death certificate data were used to identify deaths due to methadone. Linear regression was used to quantify the relationship of deaths and poison center calls.

**Results:**

Compared to decedents, poison center callers tended to be younger, more often female, at home and less likely to require medical attention. A strong association was found with PC calls and methadone mortality (b = 0.88, se = 0.42, t = 9.5, df = 1, p<0.0001, R^2^ = 0.77). These findings were robust to large changes in a sensitivity analysis assessing the impact of underreporting of methadone overdose deaths.

**Conclusions:**

Our results suggest that calls to poison centers for methadone are correlated with poisoning mortality as identified on death certificates. Calls received by poison centers may be used for timely surveillance of mortality due to methadone. In the midst of the prescription opioid overdose epidemic, electronic surveillance tools that report in real-time are powerful public health tools.

## Introduction

Increases in the prescriptive use of opioid analgesics since the early 1990s have been paralleled by an increase in medical consequences caused by their nonmedical use, as exhibited by emergency department admissions [1] and treatment seeking behavior [2]. Methadone has emerged as a commonly prescribed medication for the management of cancer and non-malignant pain [3], [4]. Simultaneously, methadone maintenance programs for the management of opioid dependence have expanded and attracted new types of patients [5]. Of concern, poisoning deaths due to prescription opioids have also risen dramatically since that time [6], [7]. Increases in methadone poisoning deaths are believed to be associated with increased use of this opioid for pain management [8], [9]. The Institute of Medicine (IOM) attributes the rise in chronic pain to an aging population, obesity, patient expectations for aggressive pain management, increased survivorship after injury, and greater numbers of surgical procedures [10].

The United States also lacks a timely, geographically-specific early warning system for opioid poisoning deaths. Reports from federally funded drug abuse monitoring systems and national vital statistics become publicly available years after events have occurred, often too late for interventions and policymaking.

All US poison centers (PCs) are capable of real-time electronic reporting, including product specific information on pharmaceutical medications, including opioid analgesics and other controlled substances [11]. These data have been used for post-marketing surveillance and evaluating the public health impact of policy changes [9], [12], [13], [14], [15], [16]. As would be expected, not all poisoning deaths are reported to PCs [17], but the application of regression models may allow PC data to be used as an early warning system for poisoning mortality and to strengthen pharmacovigilance [18], as suggested by others [19], [20], [21].

In addition to timeliness and geographic specificity, poison centers collect data on the nature of the exposure, have complete nationwide coverage, and are specific for the compound of exposure. Because of these strengths, poison centers are one component of the non-profit Researched Abuse, Diversion and Addiction Related Surveillance (RADARS®) System [22], [23], [24], [25]. Since in 2001, the RADARS System has collected surveillance data regarding the type and prevalence of prescription drug abuse, misuse, and diversion in the United States for subscribers and government agencies. The RADARS System is administered by Denver Hospitals, a non-profit research institution.

Vital statistics data have been proposed as a source for surveillance of poisoning deaths due to prescription medications [26], [27]. In this analysis, we compared death certificate data to poison center exposure calls for methadone in 29 states covered by the RADARS System from 2006 to 2007. In this manner, we were able to determine the level of association between poison center calls and overdose mortality in the United States.

## Methods

### Ethics Statement

This research project was reviewed and approved by the institutional review boards at the University of North Carolina at Chapel Hill School of Public Health and the Denver Health and Hospitals Authority, in addition to the local institutional review boards for all participating poison centers. Data were obtained in de-identified format analyzed anonymously.

### Poison Center Exposure Data

For information, identification and exposure calls, poison centers utilize a standard data collection system that includes 47 data fields, plus a notes/verbatim field. Data from any call regarding methadone were extracted from participating poison center databases. Methadone substances were defined using product-specific codes in the MICROMEDEX® Healthcare Series by Thomson Healthcare, Inc. (Greenwood Village, CO). Methadone was chosen as the drug of interest because it is the only prescription opioid that has a separate substance-level code (T40.3) in International Classification of Disease 10 Revision (ICD-10) used to identify substances implicated in poisoning deaths by the Centers for Disease Control and Prevention (CDC).

Calls to poison centers are received by nurses and pharmacists trained in toxicology. Routine data collected include demographics, substances involved, medical conditions, type and intent of exposure, the type of medical attention received when available, and the ultimate medical outcome of the case. All human intentional and unintentional exposure calls for methadone from participating poison centers from 2006 through 2007 were used for this analysis. Standard definitions for intentional, unintentional and “other” exposures, as well as standard definitions of medical outcomes were obtained from the American Association of Poison Control Centers (AAPCC) instruction manual [28]. Standard medical outcome definitions used by poison centers were included: minor effect, moderate effect, major effect, and death. Occurrences of *minor effect* were defined when the patient had minimally bothersome symptoms that resolved rapidly. *Moderate effect* was identified when patients had systemic symptoms where some type of treatment was warranted, however the effect was not life threatening. Progressively worse, *major effect*, was documented when the patient exhibited life threatening symptoms resulting from the exposure.

### Case Review & Quality Assurance of PC Data

Each call was reviewed at the respective poison center by a certified poison center specialist and reports uploaded into a database at the coordinating poison center (Rocky Mountain Poison and Drug Center, Denver, Colorado). The coding of calls was verified using the notes field to assist in evaluation, resulting in the reclassification of cases using a methodology previously described [29]. Briefly, this case review included verification of each substance, confirmation of the exposure code, and confirmation of removal of all identifying information. Inconsistencies within coding either product or exposure were corrected and the adjustment was documented.

**Figure 1:**
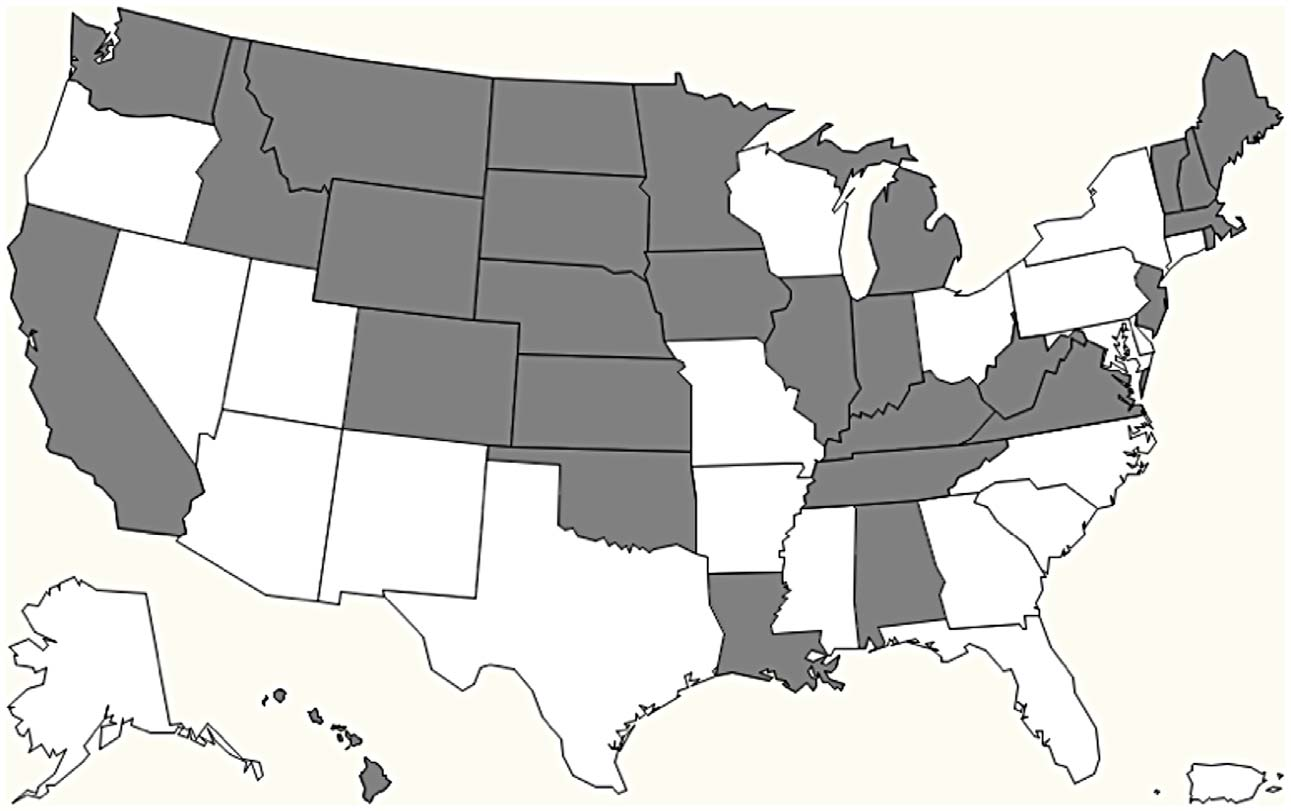
States with poison centers contributing data to study (in gray), 2006–2007. Study sites included 29 states with rural and large metropolitan areas that contributed data to the RADARS System for this analysis. These states included 49.7% of the total US population based on the 2000 Census.

RADARS System data are geocoded by the three-digit ZIP code of the location of the caller. Each poison center transfers data directly to the RADARS System; poison centers have been added as the system has expanded in geographic coverage. To account for changing coverage due to changing poison center participation in the RADARS PC program over time, only data from poison centers covering entire states in the time period were used in analysis to match the finest level of geographic specificity publicly available for US vital statistics data. Poison center calls were linked to states through reported three-digit ZIP code. This resulted in slight variations as Census data for populations use ZIP code tabulation areas, which vary slightly from postal ZIP codes used here to determine population. The populations determined by ZIP codes for covered states were similar to state populations reported in the 2000 US Census however; all estimated state populations were within 0.2% of the reported population based from ZIP code tabulation areas, suggesting a high degree of concurrent coverage between poison centers and vital statistics data. The resulting sample included data for 29 states, shown in Figure 1. Poison center coverage in these states accounted for approximately 49.7% of the total US population.

### Death Certificate Data

Deaths from unnatural causes, such as poisonings, are referred to coroners and medical examiners in accordance with state laws. Underlying and contributing causes of death are attributed after investigation, including the identification of toxic substances that were involved [30], [31]. Death certificate data are collected by state health authorities and reported to the National Center for Health Statistics (NCHS) at CDC, using ICD-10 codes. The multiple cause-of-death data files for 2006 and 2007 were obtained by special request from NCHS.

Unintentional poisonings (X42, X44), intentional poisonings (X62, X64) and poisonings of undetermined intent (Y12, Y14) were defined using the single underlying and 20 contributing causes-of-death fields. For these deaths we identified the substance(s) implicated using the toxicology (T-level) code listed as a contributing cause-of-death (T40.2 for full agonist prescription opioids, T40.3 for methadone, T40.4 for synthetic partial agonist prescription opioids); deaths due to unspecified opioids were also selected for use in sensitivity analysis (T40.6). More than one T-category of opioid could have been specified. Age, sex and place of injury were extracted. Death counts were aggregated by state over the study period for the analysis and used as the dependent variable in regression analyses.

### Regression Models & Analysis

Linear regression was used to test the association of methadone related death counts obtained from vital statistics data with counts of poison center exposure calls related to methadone obtained from the RADARS System PCs. Log transformations were utilized to meet assumptions of linearity in regression models. Initially a univariate model was constructed which included death counts as the dependent variable and poison center call counts as the predictor variable. A final regression model was constructed that included poison center call counts (as the primary explanatory variable) and population as a covariate. To further investigate the predictive potential of poison center data a cross-validation was preformed comparing data from 2006 to 2007. Predicted methadone deaths were obtained from a model of deaths regressed on calls and population from 2006 data. The 2006 predicted values were compared to actual 2007 deaths and this was assessed with a Pearson correlation coefficient and the R^2^ value is presented. The REG procedure for a linear regression using a t-statistic in SAS Enterprise Guide version 4.3 (Cary, North Carolina, USA) was used for the analysis.

### Sensitivity Analysis for Substances Unspecified

In death certificate data, the unspecified code (T40.6) is assigned if a drug substance cannot be differentially determined due to a lack of supporting evidence or ambiguous toxicology results. Some deaths in which a specific substance was not specified may have been due to methadone but not identified as such. Sensitivity analysis was conducted by running a series of models in which substance-unspecified deaths in each state were attributed to methadone in increments of 5 percent from zero to 100 percent.

## Results

Data sources and sample sizes are summarized in Table 1. Methadone was mentioned among intentional and unintentional exposure calls collected by the RADARS System poison centers in the study area 4178 times from 2006 through 2007. Based on the vital statistics data, there were 5137 decedents whose deaths were identified to have involved methadone toxicity in the study area from 2006 through 2007. This corresponded to a population rate of poison center calls regarding methadone of 1.4 per 100,000 population in 2006 and 1.6 per 100,000 population in 2007. Similarly deaths related to methadone toxicity were 1.8 per 100,000 population in 2006 and 1.8 per 100,000 population in 2007.

**Table 1:** Data sources and definitions used in analysis, 29 states, 2006–2007.

	Data Source	Population	Sample Size	Coding Scheme	Case Definition
**Vital Statistics**	CDC/NCHS,multiplecause-of-deathmortality file	Accidental and intentional methadone poisoning decedents	5137 Decedents	ICD-10	Unintentional poisoning (X42, X44) or Intentional poisoning (X62, X64) or Undetermined intent (Y12, Y14) or and Methadone toxicology (T40.3) or Unspecified narcotic (T40.6)
**Poison Centers**	The RADARS®System	Unintentional and Intentional methadone exposures, as notified to poison centers	4187 Calls	AAPCC definitions used for TESS	Intentional exposure calls, which include: Abuse Intentional misuse Intentional unknown Withdrawal Suicide

Abbreviations: Centers for Disease Control and Prevention (CDC); National Center for Health Statistics (NCHS); Researched Abuse, Diversion and Addiction Related Surveillance (RADARS) System; International Classification of Disease, 10th Revision (ICD-10); American Association of Poison Control Centers (AAPCC); Toxic Exposure Surveillance System (TESS).

### Characteristics of Exposure and Poisoning Victims

**Figure 2:**
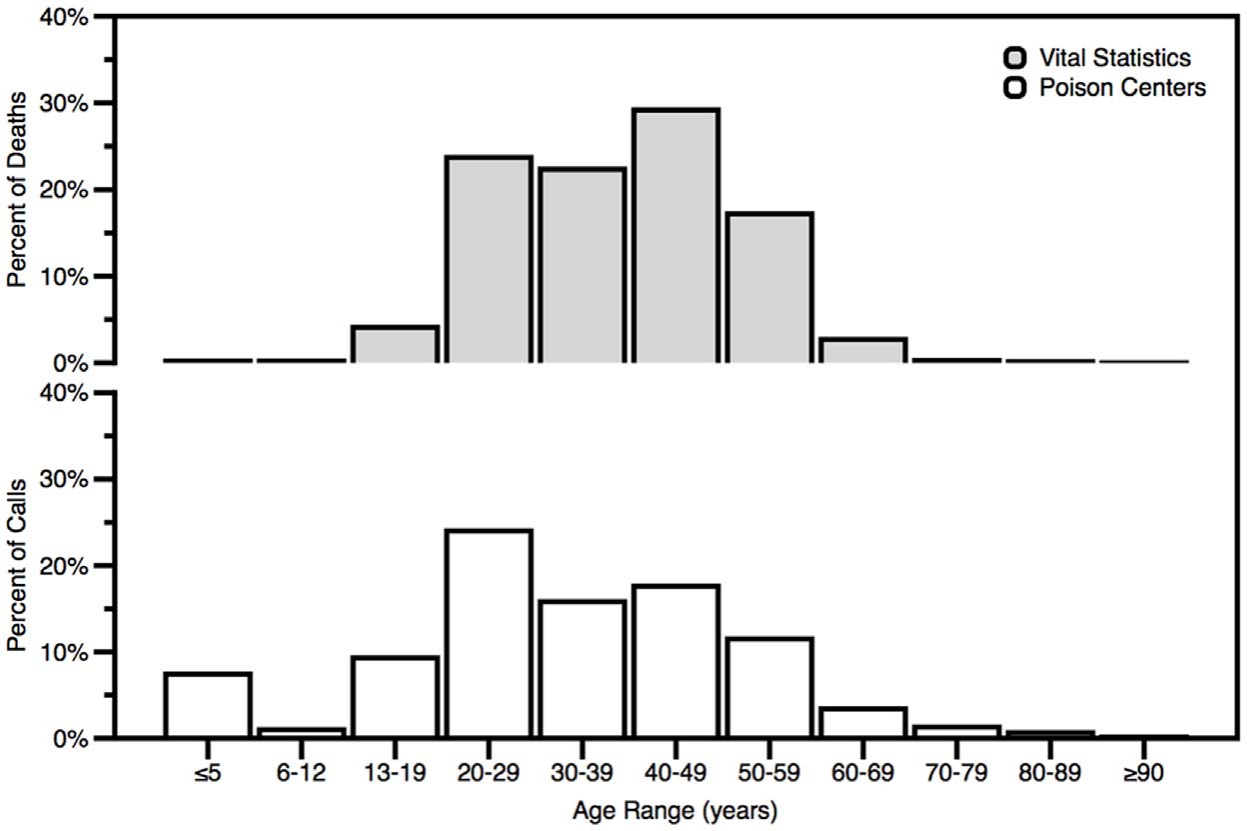
Age distributions for PC calls and poisoning deaths involving methadone, 29 states, 2006–7. Age distributions were similar, although poison center exposure calls were slightly more often about infants and children than overdose deaths reported in vital statistics. Other studies have also documented that emergency medical service utilization tends to involve younger individuals compared to overdose deaths due to prescription opioids in the United States [8].

Females comprised a higher percent of poison center calls for methadone (44.8%) than deaths from methadone poisoning (34.3%). Both poison center calls and methadone overdose deaths had bimodal age distributions, with peaks among 20–29 years and 40–49 year age groups, Figure 2. However, the peak in the earlier age group was higher among poison center calls than overdose deaths.

The site of exposure among calls to poison centers was different from the place of injury recorded on death certificates, see Table 2. Poison center exposure calls were most likely to have occurred in a healthcare facility (59.7%). Deaths due to methadone had a greater proportion of unknown/unspecified locations, and were most likely to have occurred at home (57.3%).

**Table 2:** Places of exposure/injury from methadone among poison center exposure calls and poisoning deaths, 29 states, United States, 2006–2007.

	Poison Center Exposure Calls	Poisoning Deaths
	Place of Exposure (% of sample)	Place of Injury (% of sample)
Residence	1316 (31.5%)	2941 (57.3%)
Healthcare Facility	2497 (59.7%)	1230 (23.9%)
Other/Unspecified	372 (7.8%)	966 (18.8%)

The distribution of medical outcomes of exposure for poison center calls were: no effect (8.5%), minor effect (18.8%), moderate effect (26.7%), major effect (12.1%), death (1.6%), unable to be or not followed-up (29.2%) and other (3.1%).

### The Association of Poison Center Calls and URDD with Poisoning Mortality

**Figure 3:**
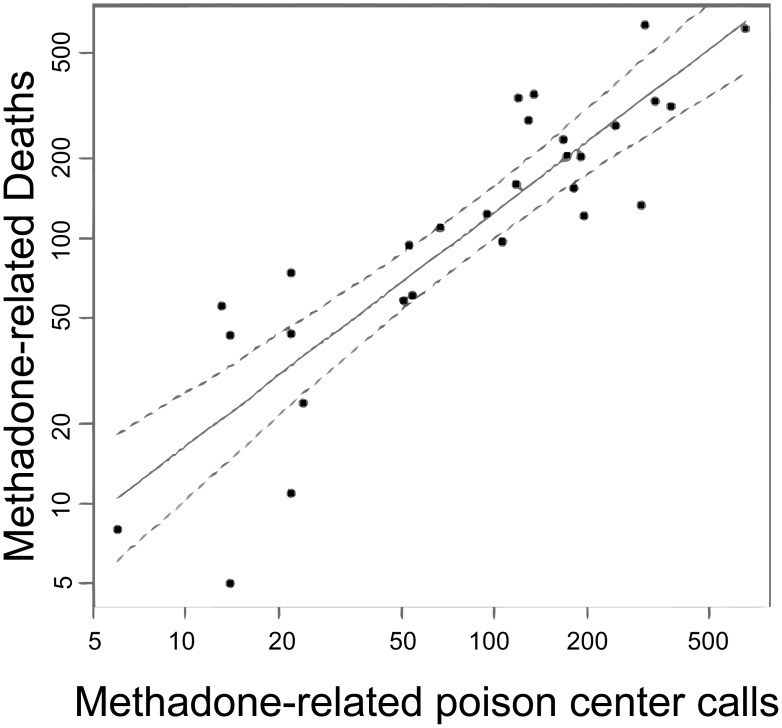
Poison center calls predict 77% of the variation in death counts from vital statistics data, 29 states, 2006–7. The plot shows the association between log-methadone death counts and log-methadone poison center calls; log scales are used for the sake of including all data on one graph since the state populations (and counts) vary widely. A strong association was found between poison center exposure calls and methadone-related mortality reported to vital statistics (b = 0.88, SE = 0.42, t = 9.5, df = 1, p<0.0001, R^2^ = 0.77), where a one unit increase of poison center calls corresponded to a 0.88 unit increase in methadone related deaths.

As seen in Figure 3, there appears to be a strong association between poison center calls and overdose deaths. In the univariate analysis; a strong association was found with PC calls and methadone mortality (b = 0.88, SE = 0.42, t = 9.5, df = 1, p<0.0001, R^2^ = 0.77), where a one unit increase of poison center calls corresponded to a 0.88 unit increase in methadone related deaths. Importantly, including population as a covariate in a regression model had nominal effect on the association of PC calls with methadone related mortality (b_adj_ = 0.71, SE = 0.18, t = 3.9 df = 1, p = 0.0006). Further cross-validation analysis suggests a high level of correlation between 2006 predicted deaths and 2007 deaths (R^2^ = 0.79).

### Sensitivity Analysis

Of deaths in our study, 17% had only one T-code classification of “narcotic unspecified” (T40.6). Some of these deaths will have been due to methadone poisoning, but would not have been accounted for in the models above. Sensitivity analysis using a univariate model revealed that the findings of this study were robust to underreported methadone deaths. Poison center calls were still associated with deaths even if all deaths due to an unspecified narcotic were recoded to be deaths due to methadone (p<0.0001, R^2^ = 0.73). As expected the association weakens somewhat, however it remains robust to misclassification of unspecified methadone related deaths.

## Discussion

In this paper we present a methodology whereby methadone poison center data can be used for surveillance of methadone poisoning deaths. Poison center calls were associated with deaths, but also could predict 77% of the variation in death counts from vital statistics data. The utility of poison center data for surveillance is further demonstrated through the association of calls with deaths, even after normalization for underlying population.

This study builds on the work of previous investigations of the utility in using poison center data for surveillance of nonmedical use of and adverse consequences due to nonmedical use of prescription opioids [32], [33], [34]. Similar studies have been conducted for nonprescription medications [35], [36], [37], [38], [39]. Our primary finding is that poison center data may be used as a predictive tool for methadone poisoning deaths, due to their more rapid availability than national vital statistics data. Previous studies on the utility of poison center data for poisoning surveillance have examined whether deaths reported in TESS appear in vital statistics reports of mortality [17], [40], [41], [42], [43]. However, our results suggest that exposure calls, regardless of the disposition of the patient, can serve as an early warning system for overdose deaths. Due to the ecologic nature of this analysis, we cannot make a causal connection between poison center exposure calls and deaths. We suggest our results be interpreted as an early warning system for situational awareness.

Exposure call victims reported to poison centers were younger and more often female than those who died from methadone poisonings. One possible explanation of these differences includes that older poisoning victims may have comorbid conditions that decrease their likelihood for surviving respiratory depression. Sims et al. compared methadone-related adverse events in Utah that were reported to emergency departments, the office of the medical examiner, state vital statistics and a controlled substance prescription database [8]. They found increases in methadone-related mentions or prescriptions in all four reporting systems between 1997 and 2004; however, they also noted distinct differences in age, sex and urbanicity of place of residence of individuals reported to each reporting system. Females were also overrepresented in their emergency department sample, compared to decedents listed in vital statistics. In their study ED patients were older than the poison center exposure calls, suggesting further work is needed to characterize who accesses medical care for poisonings through which avenue, so that services can be better targeted to fit the needs of the corresponding demographics.

More than a quarter of calls (no effect and minor effect, 27.3%) received did not warrant medical attention, yet the exposed individual or someone around them considered the exposure serious enough to dial a poison center. This may suggest that there are idiosyncratic thresholds for seeking help for methadone exposure. It also raises the question of what level of effect would be considered an “overdose” in retrospective self-reported questionnaires.

We acknowledge the following limitations of our study. First, the inclusion of only RADARS System poison centers may affect the generalizability of our findings. The 29 states represented in this analysis include heterogeneous areas including some of the largest metropolitan areas in the country, as well states with considerable rural population. Collectively, nearly half of the United States population was covered by the poison centers in this analysis. Second, we cannot tell from these data alone whether the observed association also holds for prescription opioids other than methadone. Limitations in the ICD-10 coding schema make it difficult to assess overdose deaths involving other prescription opioids in our framework. Adding in other prescription opioids to the analysis would have introduced a level of pharmacological heterogeneity that would limit interpretation of the findings. For example, fentanyl, buprenorphine and tramadol are all included in the same category (T40.4), despite markedly different toxicological profiles (respiratory depression from a full mu-receptor agonist, polysubstance toxicology risk with a partial agonist-antagonist, and serotonin depletion, respectively). Third, death certificate data for poisonings have known shortcomings that we have previously articulated [31], [44], [45]. One concern is the classification of intent of death [46], [47]. In order to avoid these shortcomings we chose to include all deaths with a toxicology code for methadone, including unintentional and intentional poisonings. In doing so we may have introduced a level of heterogeneity. A review of the underlying cause-of-death codes revealed that the great majority of methadone poising deaths were due to accidental poisonings. Similar results were seen in the poison center data. However, it remains a question whether the methods presented in this analysis are better suited for unintentional or intentional poisonings, or both.

### Conclusion

Ubiquitous coverage, timeliness, geographic specificity, availability and quality make poison center data attractive for surveillance purposes. In this paper we presented methods for making use of poison center data to anticipate methadone poisoning deaths. RADARS System poison center exposure calls show correlation with poisoning mortality for methadone in 29 states in 2006 through 2007, with an 88 percent increase in deaths per unit increase in PC exposure calls. Further work needs to be done in characterizing and understanding the nature of poison center exposure calls, in order to better utilize this data source.
